# Pathological Mechanism for Delayed Hyperenhancement of Chronic Scarred Myocardium in Contrast Agent Enhanced Magnetic Resonance Imaging

**DOI:** 10.1371/journal.pone.0096463

**Published:** 2014-05-06

**Authors:** Jian Wang, Bo Xiang, Hung-Yu Lin, Hongyu Liu, Darren Freed, Rakesh C. Arora, Ganghong Tian

**Affiliations:** 1 Department of Vascular Surgery, Union Hospital, Tongji Medical College, Huazhong University of Science and Technology, Wuhan, Hubei, China; 2 National Research Council, Winnipeg, Manitoba, Canada; 3 Department of Physiology, Faculty of Medicine, University of Manitoba, Winnipeg, Manitoba, Canada; 4 Department of Cardiac Surgery, the First Affiliated Hospital, Harbin Medical University, Harbin, Heilongjiang, China; 5 Cardiac Science Program, Institute of Cardiovascular Sciences, St. Boniface General Hospital, Winnipeg, Manitoba, Canada; Scuola Superiore Sant'Anna, Italy

## Abstract

**Objectives:**

To evaluate possible mechanism for delayed hyperenhancement of scarred myocardium by investigating the relationship of contrast agent (CA) first pass and delayed enhancement patterns with histopathological changes.

**Materials and Methods:**

Eighteen pigs underwent 4 weeks ligation of 1 or 2 diagonal coronary arteries to induce chronic infarction. The hearts were then removed and perfused in a Langendorff apparatus. The hearts firstly experienced phosphorus 31 MR spectroscopy. The hearts in group I (*n* = 9) and II (*n* = 9) then received the bolus injection of Gadolinium diethylenetriamine pentaacetic acid (0.05 mmol/kg) and gadolinium-based macromolecular agent (P792, 15 µmol/kg), respectively. First pass T_2_^*^ MRI was acquired using a gradient echo sequence. Delayed enhanced T_1_ MRI was acquired with an inversion recovery sequence. Masson's trichrome and anti- von Willebrand Factor (vWF) staining were performed for infarct characterization.

**Results:**

Wash-in of both kinds of CA caused the sharp and dramatic T_2_^*^ signal decrease of scarred myocardium similar to that of normal myocardium. Myocardial blood flow and microvessel density were significantly recovered in 4-week-old scar tissue. Steady state distribution volume (ΔR_1_ relaxation rate) of Gd-DTPA was markedly higher in scarred myocardium than in normal myocardium, whereas ΔR_1_ relaxation rate of P792 did not differ significantly between scarred and normal myocardium. The ratio of extracellular volume to the total water volume was significantly greater in scarred myocardium than in normal myocardium. Scarred myocardium contained massive residual capillaries and dilated vessels. Histological stains indicated the extensively discrete matrix deposition and lack of cellular structure in scarred myocardium.

**Conclusions:**

Collateral circulation formation and residual vessel effectively delivered CA into scarred myocardium. However, residual vessel without abnormal hyperpermeability allowed Gd-DTPA rather than P792 to penetrate into extravascular compartment. Discrete collagen fiber meshwork and loss of cellularity enlarged extracellular space accessible to Gd-DTPA, resulting in the delayed hyper-enhanced scar.

## Introduction

MR delayed hyperenhanement after administration of extracellular contrast agent (CA) such as Gd-DTPA was exclusively associated with scar tissue in chronic myocardial infarction (MI) [1], [2]. The accuracy of delayed enhancement imaging in determining scar size was validated with nuclear imaging techniques [3], [4]. However, the mechanism for delayed hyperenhancement of scarred MI remains poorly understood. Scar healing after MI is associated with vascular adaption and structural reconfiguration, greatly affecting first pass and delayed distribution of CA. Therefore, to investigate the relationship of CA first pass and delayed enhancement patterns with pathological changes might provide important insight into possible mechanism.

MR delayed enhancement observed after CA injection is affected by the infarct-related artery patency and microvascular obstruction [5], [6], [7]. After release of prolonged coronary occlusion, restoration of blood flow to infracted regions is heterogeneous and relevant to differential delayed enhancement patterns in acute reperfused MI. Hyperenhanced regions, mainly located in the periphery of acute MI, display definite restoration of regional blood flow [5], [6]. After massive CAs enter into MI region with effective reperfusion, loss of cell membrane integrity cannot exclude CA from intracellular space and results in an enlarged distribution volume. Hypoenhancement at the infarct core is closely related to the no-reflow phenomenon, which reveals severe and widespread microvascular obstruction due to erythrocytes stasis, neutrophils accumulation and endothelial cell swelling [7], [8]. Consequently, effective delivery of CA into MI regions is the prerequisite for late delayed hyperenhancement. Whether the reestablishment of collateral circulation during scar healing allows for CA into scarred myocardium remains to be clarified.

Equilibrium distribution patterns of CA not only depend on its delivery by blood flow perfusion, but also on the properties of CA, vascular permeability and the size of interstitial space accessible to CA. Myocytes necrosis results in the disruption of cellular membrane, allowing for extracellular CA into intracellular space and creating an increased distribution volume [9], [10]. Therefore, myocardial cell injury might be evaluated by extracellular agent. Additionally, myocardial ischemia causes the injury to vasculature and elevates the permeability of capillaries. Intravascular agent is retained within intravascular space in normal myocardium, but leaks into interstitial space and causes a relatively increased distribution volume in MI region [11], [12]. Therefore, microvascular damage might be probed by intravascular agent. Scar repairing in chronic MI originates from complete cellular necrosis and ends with marked deposition of collagen matrix. It is unclear whether vascular reconstruction causes hyperpermeability of vessel wall in scar tissue. Therefore, the potential of MRI to provide information about vascular permeability and the accessibility of interstitial space will be determined by the relation of contrast enhancement patterns with the underlying pathophysiology.

Phenylphosphonate (PPA) distributes only into the extracellular compartment, whereas dimethyl methylphosphonate (DMMP) evenly expands into the entire water space. Both phosphorous-containing compounds have the specific and distinct peak position in phosphorous 31 MR spectra, and intensity of corresponding peak reflects the volume of extracellular compartment and the entire water space. Thus, the ratio of PPA to DMMP signal intensity can reflect the percentage of interstitial compartment to the whole space.

## Materials and Methods

### Ethics statement

This study was carried out in strict accordance with the recommendations in the Guide for the Care and Use of Laboratory Animals of Huazhong University of Science and Technology. The protocol was approved by the Committee on the Ethics of Animal Experiments of Wuhan Union Hospital (Permit Number: 2008037). All surgery were performed under general anesthesia, and all efforts were made to minimize suffering.

### Pig model of chronic scarred myocardium

Eighteen domestic pigs weighing 20–25 kg were sedated with an intramuscular injection of diazepam (0.4 mg/kg body wt) and ketamine (20 mg/kg body wt). After the induction of anesthemia, pigs were intubated and ventilated with gas anesthesia comprising 1%–2% isoflurance in a mixture of oxygen and nitrous oxide. Using a sterile technique, a lateral thoracotomy was performed at the fourth left intercostals space to expose the heart, and the pericardium was opened. The first and second diagonal branches of left anterior descending coronary artery (LAD) were permanently ligated at their origin. The presence of occlusion was confirmed by development of regional myocardial cyanosis. The chests were closed and the animals were allowed to recover for 4 weeks for the development of chronic scarred myocardium.

### Isolated pig heart preparation

At the end of 4-week recovery period, animal chest was reopened through median sternotomy under a general anesthesia. The pericardium was opened longitudinally along the midline. The aorta and pulmonary artery, inferior and superior vena cava were dissected and clamped. Cold (−4°C) cardioplegia was infused into aortic root to arrest the heart. The heart was quickly excised and immersed in cold saline solution for instrumentation. The brachiocephalic artery and subclavian artery were cannulated for achievement of antegrade perfusion and measurement of antegrade pressure. A mixture of autogenous blood and modified Krebs-Henseleit solution in 1∶1 ratio was used to perfuse the hearts. The concentrations of potassium and magnesium in the perfusion medium were each adjusted in 16 mmol/l to keep hearts quiescent throughout the imaging period.

### Experimental protocol

Isolated hearts were placed in Langendorff perfusion apparatus positioned in the center of a 7T magnet. The hearts were perfused by mixture of pig blood with K-H solution at perfusion pressure of approximately 60 mmHg. All hearts were subjected to a protocol consisting of 40-min P^31^ MR spectroscopy and 55-min acquisition of MR imaging. Dimenthyl methylphosphonate (DMMP, 20 mmol/L) and phenylphosphonic acid (PPA, 10 mmol/L) were added into perfusion medium. ^31^P MR spectra were acquired from normal region and scarred myocardium, respectively. DMMP evenly distributes to the entire water space, whereas PPA distributes only into extracellular compartment. Both phosphorous-containing compounds have the specific peak position in ^31^P MR spectra, and intensity of corresponding peak reflects the volume of extracellular compartment and the entire water space.

In the phase of MR imaging studies, T_1_- weighted imaging was firstly performed without CA using T_1_-weighted inversion-recovery (IR) turbo fast low-angle shot (turboFLASH) sequence. Afterward, hearts received the bolus injection of either 0.05 mmol/kg of Gd-DTPA (group I; n = 9) or 15 µmol/kg of P792 (group II; n = 9), respectively. To prevent air-tissue interface artifact on T_2_^*^ - weighted imaging, the pig heart was immersed in the perfusate during images study. The effect of CA on T_2_^*^ signal intensity during its first passage through myocardium was monitored using a gradient-recalled echo (GRE) fast low-angle shot (FLASH) sequence. The CA delayed enhanced T_1_ imaging was conducted at 5 different time points with 10-minute time interval after CA administration with a T_1_-weighted IR turboFLASH sequence.

Following the MR imaging studies, perfusion was maintained as during MR imaging and the hearts were injected with colored microsphere to assess regional myocardial blood flow. Hearts were removed from perfusion line and sectioned into slices according to scout imaging to ensure a correlation to MR imaging. The slices were used to perform microsphere analysis and histological examination,

### Colored microspheres and histochemical staining procedure

At the end of each experiment, approximately 5×10^6^ to 8×10^6^ nonradioactive red-colored microspheres (15±1.9 µm diameter, suspended in 2 ml of saline solution) were injected through the aorta. Arterial reference samples were withdrawn simultaneously from the ascending aorta at a constant rate of 5 ml/mm for 3 min, starting 1 min before injection of the microspheres. Hearts were removed and sectioned into short axis slice from base to apex. Alternating sections were used for histochemical staining, with the opposing side used for colored microsphere analysis. Sections for histochemical staining firstly were stained by 2% TTC solution and photographed under room light to determine the infarct region. Following TTC staining, there sections were separated into the infracted scar tissue and normal myocardium, which were frozen by immersion in liquid nitrogen. 5 µm-thick slices from two kinds of myocardium were stained with hematoxylin and eosin. Massion's trichrome stain was used to define scarred myocardium.

Pie – shaped sample was collected from normal regions and infarct scar. Positive TTC staining in the matched, opposing sections and thinning of ventricular wall can be thought to be the index of infarcted region. The average weight of each sample was approximately 0.5 g. After collection, tissue and blood sample were digested. The numbers of microsoheres in the samples were counted using a spectrophotometer at a wavelength of 536 nm. Actual blood flow was calculated based on the microsphere count in the tissue and blood samples and speed of blood collection.

### Immunohistochemical staining for von-Willebrand Factor (vWF)

The endothelial cell and angiogenesis were traced by antibody to vWF (Sigma, St. Louis, MO, USA). Tissue sections were fixed with 4% paraformaldehyde for 10 minutes at room temperature, washed three times with PBS containing 0.3% Triton X-100, and blocked with 2% goat serum and 1% bovine serium albumin (BSA) for 30 minutes at room temperature. Slides were then incubated with primary antibodies against vWF for 1 hour at 37°C. After three washes with PBS, slides were incubated with Alexa Fluor 594-conjugated goat anti pig IgG (Invitrogen, Invitrogen, Carlsbad, CA, USA) for 45 minutes at 37°C. After three more washes with PBS, the slides were stained for nuclei with 4′,6-diamidino-2-phenylindole (DAPI, Sigma). The slides were observed by a Carl Zeiss fluorescence microscope. Microvessels were counted in at least three random fields under a fluorescence microscope. The vWF-positive capillaries were counted and the microvessel density was expressed as counts per mm^2^.

### ^31^P MR spectroscopy

^31^P MR spectroscopy was performed on a 7-Tesla magnet equipped with a Biospec spectrometer (Bruker, Karlsruhe, Germany). A MR surface coil with 1.5 cm of diameter was firstly positioned over chronic scarred region. Then the surface coil was moved to normal myocardium of LAD region. The P^31^ MR spectra were therefore acquired from normal myocardium and infarcted scar region, respectively.

MR signals (free induction decay, FID) were obtained by use of a hard pulse with a pulse length of 75 µsec and a repetition time of 2 sec. Six FID signals were accumulated for each ^31^P MR spectrum. Thus each spectrum was averaged over a 2-minute sample time.

The observed phosphorus compounds included DMMP, PPA, inorganic phosphate (Pi), phosphocreatine (PCr), three peaks (α, β, γ) of adenosine triphosphate (ATP).

### Magnetic resonance imaging

MR imaging studies were performed on a similar 7T, 40 cm horizontal bore magnet. MR images were acquired with a Helmholtz coil. Scout images were acquired to locate long axis, from which three parallel short-axis slices were prescribed.

Monitoring of T_2_^*^ signal intensity was performed using a FLASH gradient echo sequence during the first pass of CA. Gradient echo sequence parameters were as follows: matrix: 128×128; field of view: 120×120 mm; slice thickness: 3 mm; flip angle: 8°, echo time: 15mesc; acquisition time: 1815.16msec; repetition time: 26msec. All MR images were acquired from the arrested heart to eliminate motion artifact.

Regular T_1_-weighted images were acquired with a TurboFLASH inversion-recovery sequence. Nine T_1_-weighted images were obtained with nine different inversion times (10, 100, 200, 400, 700, 1200, 2000, 5000 and 10,000msec) to measure T1 relaxation time and calculate ΔR_1_ relaxation rate. All T_1_ images were acquired from a 5 mm thick slice with a 120×120 mm field of view and imager matrix size of 128×128.

### Data analysis

^31^P spectra were analyzed using 1D-WINNER (Bruker, Karlsruhe, Germany). The ratio of extracellular volume to the entire water volume was obtained from dividing DMMP intensity peak by PPA intensity peak. MR imaging data were processed using Marevisi software package (NRC-Institute for Biodiagnostics, Winnipeg, Canada). Round, hand-drawn regions of interest (ROIs) were created by correlating the MR images to the TTC-stained references.

T_2_^*^ time intensity curves were obtained from 50 consecutive perfusion images using the Marevisi software. For one slice, two curves for scarred and normal myocardium were generated to represent the characteristic passage of CA. T_1_ relaxation time with or without CA was measured according to T_1_ imaging acquired with nine different inversion time in scarred and normal myocardium. Distribution volume of CAs was expressed as ΔR_1_ relaxation rate (1/T_1_ after contrast administration −1/T_1_ before contrast administration).

### Statistical analyses

All numerical results were presented as mean ± standard deviation. Statistical analysis was performed using Statistica (Statsoft Inc, Tulsa, OK). Differences in signal intensity and ΔR_1_ relaxation rate between normal and scarred myocardium at specific time points were analyzed by Bonferroni's *t* test. The changes over time of signal intensity and ΔR_1_ relaxation rate obtained from scarred myocardium were compared with the patterns obtained from normal myocardium by repeated measures analysis of variance (ANOVA). Moreover, differences in these values determined by histology and spectroscopy were studied by means of a paired *t* test. A value of *p*<0.05 indicates statistical significance.

## Results

### First pass kinetics and MR perfusion imagings of extravascular or intravascular CA

**Figure 1:**
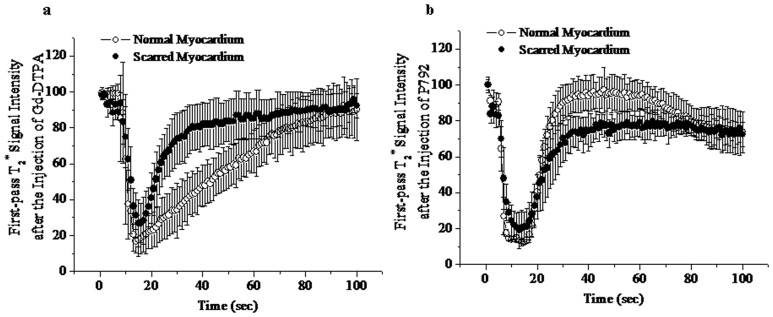
T_2_^*^ time intensity curves obtained during the first pass of Gd-DTPA (a) and P792 (b). Wash-in of both contrast agents caused similar decrease in T_2_^*^ signal intensity of scarred and normal myocardium.

**Figure 2:**
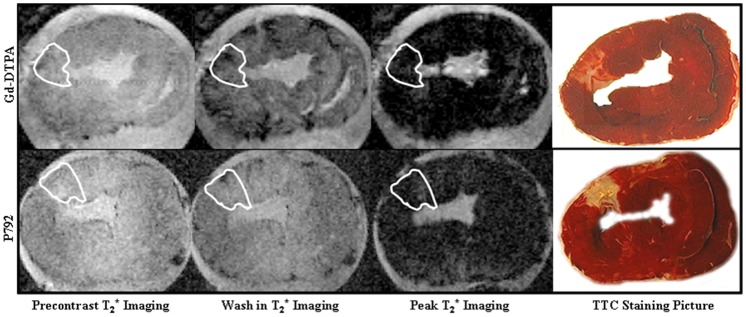
Representative contrast agent first pass T_2_^*^ imagings with a photomicrograph of TTC-stained tissue. The extent of T_2_^*^ signal hypoenhancement caused by entry of Gd-DTPA and P792 in scarred myocardium were comparable to those in normal myocardium. There were no differences in T_2_^*^ signal loss between scarred and normal myocardium at wash-in and peak T_2_^*^ imaging. Upper panel: T_2_^*^ imaging during the first pass of Gd-DTPA; Lower panel: T_2_^*^ imaging during the first pass of P792; First vertical line: precontrast T_2_^*^ imaging; Second vertical line: wash-in T_2_^*^ imaging; Third vertical line: peak T_2_^*^ imaging; Fourth vertical line: TTC stained section.

Baseline T_2_^*^ signal intensity (SI) of scarred and normal myocardium did not differ significantly: 98.2%±2.5% versus 99.2%±3.1% of initial intensity. Wash-in of Gd-DTPA resulted in a great extent of T_2_^*^ signal decrease in scarred myocardium (from 98.2%±2.5% to 23.7%±13.1%) (Figure 1a), even though peak T_2_^*^ SI in scarred myocardium was higher than that in normal myocardium (23.7%±13.1% versus 17.8%±6.8%). Moreover, there wasn't time difference in peak effect or decline of T_2_^*^ SI between scarred and normal myocardium (Figure 1a). Therefore, the extent of T_2_^*^ signal hypoenhancement in scarred myocardium was equivalent to that in normal myocardium at T_2_^*^ wash-in and peak imagings of Gd-DTPA (Upper Panel, Figure 2).

The magnitude of T_2_^*^ signal loss caused by wash-in of P792 in scarred myocardium was comparable to that in normal myocardium (Figure 1b). No delayed transit was observed in scarred myocardium. Thus, there was not difference in T_2_^*^ signal decrement between normal and scarred myocardium at T_2_^*^ wash-in and peak imagines of P792 (Lower Panel, Figure 2). These suggest that low and high molecular weight CAs both can be effectively delivered into scarred myocardium.

### The ratio of extracellular volume to the entire water space in normal and scarred myocardium

**Figure 3:**
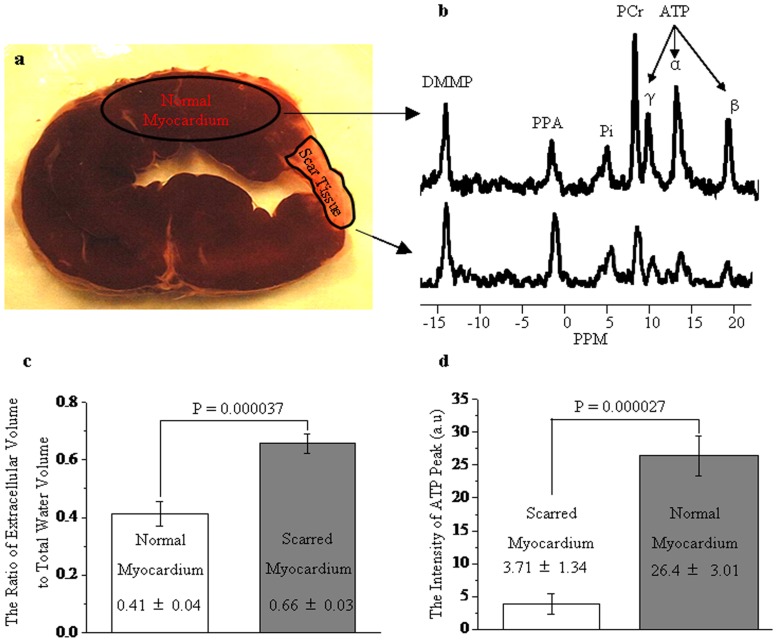
Representative ^31^P spectra acquired from scarred and normal myocardium. ^31^P spectra from scarred myocardium exhibited a more striking PPA peak (the extracellular compartment) as compared with normal myocardium (a and b). The ratio of extracellular volume to the entire space was significantly greater in scarred myocardium than in normal myocardium (c). The ATP content was substantially lower in scarred myocardium than in normal myocardium (d).

Normal myocardium was stained brick red, whereas scarred myocardium wasn't completely stained by TTC (Figure 3a). ^31^P spectra acquired from scarred myocardium displayed a more striking PPA peak and a negligible ATP peak relative to normal myocardium (Figure 3b). The ratio of extracellular volume to the entire water volume was significantly greater in scarred myocardium (0.66±0.03) than in normal myocardium (0.41±0.04) (Figure 3c). These suggest that extracellular volume is significantly enlarged in scarred myocardium. The ATP content was 26.4±3.01 a.u in normal myocardium, whereas scarred myocardium contained almost no ATP (3.71±1.34 a.u) (Figure 3d). This further demonstrates that ^31^P spectra were exclusively obtained from scarred myocardium.

### Distribution volumes and delayed enhanced MR imagings of extravascular and intravascular CA

**Figure 4:**
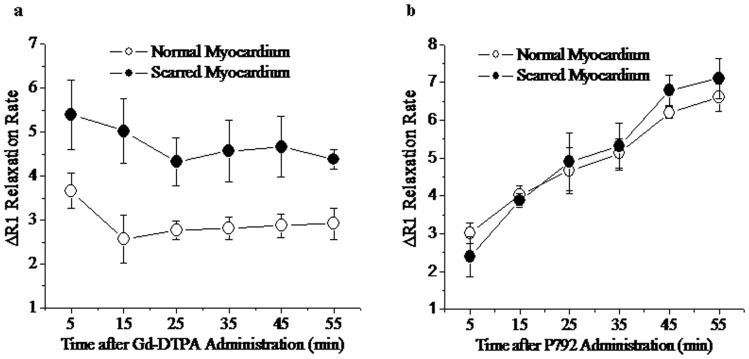
ΔR1 relaxation times measured after the administration of Gd-DTPA (a) and P792 (b). Administration of Gd-DTPA was associated with a higher ΔR1 relaxation time of scarred myocardium compared to normal myocardium, whereas there was not difference in ΔR1 relaxation time between scarred and normal myocardium post injection of P792.

**Figure 5:**
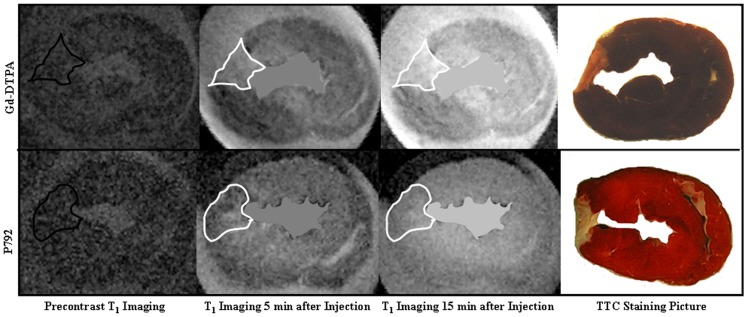
Representative contrast agent delayed enhanced T_1_ imagings with a photomicrograph of TTC-stained tissue. Scarred myocardium exhibited the hyperenhancement at Gd-DTPA enhanced T_1_ imaging, whereas homogeneous hyperenhancement was observed in scarred and normal myocardium after the administration of P792. Upper panel: Gd-DTPA enhanced T_1_ imaging; Lower panel: P792 enhanced T_1_ imaging; First vertical line: precontrast T_1_ imaging; Second vertical line: T_1_ imaging 5 min post injection; Third vertical line: T_1_ imaging 15 min post injection; Fourth vertical line: TTC stained section.

After the administration of Gd-DTPA, the ΔR_1_ relaxation rates of scarred myocardium ranged from 5.4±0.7 to 4.3±0.2 for different time points postinjection of Gd-DTPA and were significantly greater than those of normal myocardium, ranging from 3.6±0.4 to 2.9±0.3 (Figure 4a). Therefore, scarred myocardium exhibited the significant hyperenhancement compared with normal myocardium in Gd-DTPA enhanced T_1_ imaging (Upper Panel, Figure 5). These suggest that expanded extravascular compartment is accessible to Gd-DTPA in scarred myocardium.

After the administration of P792, the ΔR_1_ relaxation rates of scarred myocardium were not significantly different from those of normal myocardium for different time times post injection of P792 (Figure 4b). Thus, homogeneous hyperenhancement was observed within scarred and normal myocardium in P792-enhanced T_1_ imaging (Lower Panel, Figure 5). These suggest that capillary wall integrity in scarred myocardium exclude P792 from enlarged extravascular compartment.

### Staining of histological sections

**Figure 6:**
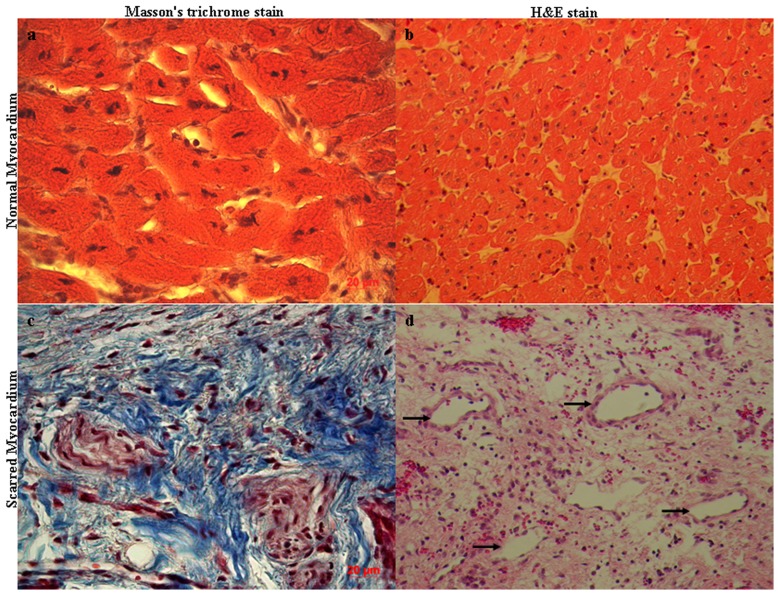
Histological staining sections: normal myocardium (upper panel) and scarred myocardium (lower panel), Masson's trichrome stain (right panel) and H&E stain (left panel). Cadiomyocytes are the major structural elements and interstitial volume only account for a small part of extravascular space of normal myocardium (a and b). The discrete compartment of collagen fiber is major framework of extracellular space in scarred myocardium (c). Cadiomyocytes have almost disappeared and vessels of different sizes were seen frequently (d).

Cardiomyocytes were major structural elements and limited extracellular space was accessible to CA due to intact cell membrane (Figure 6a and 6b). Cardiomyocyes had almost vanished and were displaced by extensively extracellular collagen matrix in scarred myocardium (Figure 6c), while vessels of different sizes were seen frequently (Arrowheads, Figure 6d).

Dilated vessels detected within scar tissue provide important routes by which effective delivery and even distribution are rapidly achieved following the injection of CAs. Formation of fibrosis collagen meshwork and loss of cadiomyocytes enlarge extravascular compartment accessible to extracellular agents, leading to the increased distribution volume in scarred myocardium.

### Myocardial blood flow and microvessel density

**Figure 7:**
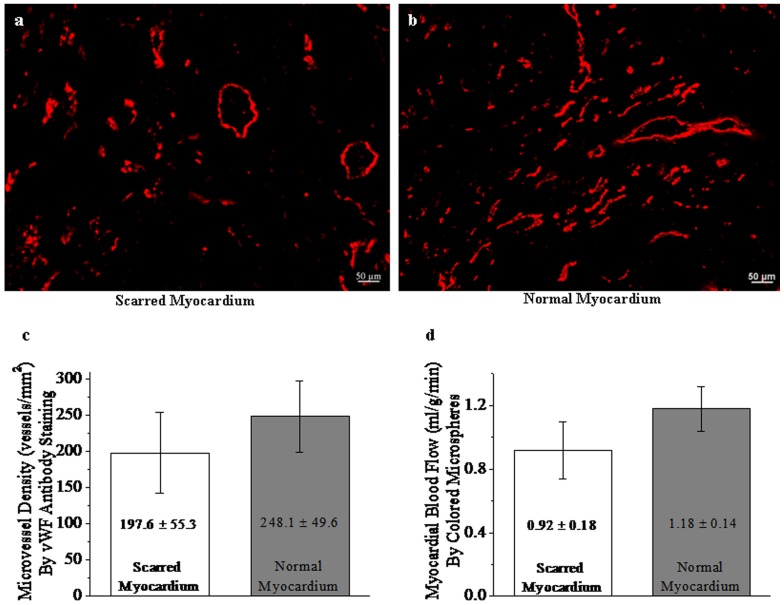
Myocardial blood flow and microvessel density. Scarred myocardium exhibited the dilated vessels and numerous microvessels (a), which resembled those observed in normal myocardium (b). Myocardial blood flow and microvessel density were significantly recovered to 0.92±0.18 ml/g/min (c) and 197.6±55.3 vessels/mm^2^ (d) despite slightly lower than these observed measured in normal myocardium.

Anti-vWF staining of scar tissue showed large blood vessels and numerous micorvessels (Figure 7a), which resembled those observed in normal myocardium (Figure 7b). Myocardial microvessel density was substantially recovered to 197.6±55.3 vessels/mm^2^, reaching approximately 79.6% of that in normal myocardium (248.1±49.6 vessels/mm^2^) (Figure 7c).

The microsphere technique also demonstrated that myocardial blood flow to scarred myocardium was significantly restored to 0.92±0.18 ml/g/min, which was slightly lower than that in normal myocardium (1.18±0.14 ml/g/min) (Figure 7d). Therefore, myocardial blood flow and microvessel density have developed rapidly to a surprising level after 4 weeks post infarction.

## Discussion

Extracellular and intravascular CAs have dissimilar molecular weights (562 versus 6473 D) and diameters (0.9 versus 5 nm) determining different distribution volume. Thus, we investigated the first pass and delayed enhancement patterns with both types of CAs in chronic MI. Histoimmunopathological examination was performed to characterize scarred myocardium. The percentage of extracellular compartment to the whole water space was evaluated using ^31^P MR spectroscopy with DMMP and PPA. Restoration of blood flow and alteration on interstitial space in scarred myocardium were compared with those in normal myocardium to define the differential contrast enhancement patterns. All findings demonstrated that pathological changes of scarred myocardium laid structural foundation for substantial wash-in of CAs during first pass and delayed hyperenhancement during following equilibrium.

Entry of CAs into myocardium creates a heterogeneous gradient across capillary wall, resulting in the loss of T_2_^*^ SI. After the rapid bolus of both kinds of CAs, rapid and dramatic T_2_^*^ signal decline was observed in scarred and normal myocardium. The extent of T_2_^*^ signal loss in scarred myocardium was almost equivalent to that in normal myocardium. There were not differences in time to peak (time interval between preinjection and the peak of bolus injection) between scarred and normal myocardium. No perfusion defect was detected on first-pass MRI in 4-week-old scar infarction, which is consistent with prior study demonstrating that the first-pass perfusion defect is detected 4 hours after permanent coronary occlusion and the perfusion defect disappeared 8 days afterwards [13]. These provide strong evidences that two types of CAs can be effectively delivered into scarred myocardium.

We found that myocardial blood flow and microvessel density of scarred myocardium had developed to a surprising level at 4 week postinfarction. Regional myocardial blood flows were 0.92±0.18 and 1.18±0.14 in scarred and normal myocardium, respectively. Myocardial microvessel densities were 197.6±55.3 and 248.1±49.6 vessels/mm^2^ in scarred and normal myocardium, respectively. Other investigators also have demonstrated that the pig develops a significant collateral network and exhibits a substantial increase in collateral blood flow after 3–4 weeks of gradual coronary artery occlusion [14], [15], [16]. Similar animal model as our study displayed that collateral blood flow was increased dramatically at 4 weeks after permanent coronary occlusion compared to the onset of acute occlusion [17]. Clinical human study also have indicated that well-developed collateral vessels are rarely present at the time of infarction and are manifest with the well visualization of vessel distal to occlusion in angiogram within 2 weeks after permanent occlusion of diseased artery [18]. Therefore, the gradual establishment of collateral circulation might be direct reason for the buck of CAs to flow into scarred myocardium. Coronary collateral vessels originated principally from the branches of other coronary artery or extracardiac artery mainly involving the internal mammary arteries and the bronchial arteries [16]. Maturation and growth of collateral vessel serve as the chief avenue from the other coronary artery to scarred myocardium, typically, bridging across the occluded coronary artery. In addition, our histological staining of scar tissue showed that dilated vessels could be seen frequently within the infracted scar tissue. Although the bulk of capillaries of infracted myocardium disappeared due to ischemic necrosis, many dilated vessels seemed to persist in scarred myocardium [19].

The relative distribution volume of CAs in the tissue can be estimated from the relative change (Δ) in the longitudinal relaxation rate (R_1_) (ΔR_1_ = 1/T_1_ before contrast administration - 1/T_1_ after contrast administration) [11], [20]. We found that ΔR_1_ relaxation rate of Gd-DTPA (small molecular weight) was significant higher in scarred myocardium than in normal myocardium. This observation is in agreement with several prior studies. Flacke et al demonstrated that the distribution coefficients (λ) of Gd-based contrast in chronic infarction were significantly elevated as compared with those in normal myocardium [21]. Rehwald et al demonstrated that Gd-DTPA concentrations were increased in the infracted scar tissue in comparison with normal myocardium using electron probe x-ray microanalysis [22]. Conversely, there was not a significant difference in ΔR_1_ relaxation rate of P792 (large molecular weight) between scarred and normal myocardium. Saeed et al also found that intravascular CA did not produce the delayed hyperenhancement of 8-week-old scar tissue [23]. The absence of delayed enhancement in chronic infarctions after intravascular CA can be attributed to the intact permeability of remodeled vessel which retains intravascular contrast. More importantly, ^31^P spectra in conjunction with chemical marker of cellular compartment (DMMP and PPA) found that ratio of extracellular volume to the entire water volume was significant higher in scarred myocardium than in normal myocardium. Therefore, delayed enhancement of scar should be attributed to the increased extracellular volume percentage and the enlarged distribution volume of Gd-DTPA.

Striking reduction in cellularity enlarges percentage of the extracellular volume in scarred myocardium. Permanent coronary occlusion produces sudden and total interruption of blood and oxygen supply to cardiomyocytes, resulting in extensive cardiomyocytes necrosis achieved by coagulation necrosis and myocytosis [24]. As a result, histological section of scarred myocardium demonstrated that cardiomyocytes had almost completely vanished at 4 week post infarction. Myocytes density declines abruptly from 98% preinfarction to 9.8% after 1 week and 2.7% after 3 weeks post-infarction in the infracted region [19]. Additionally, extensively extracellular matrix deposition is believed to be another critical reason for the increased percentage of extracellular volume. Fibrillar collagen content in the developing scar tissue increases steadily over the first few weeks after myocardial infarction, reaching a plateau after 4 weeks or more post-infarction [25], [26]. Subsequently, accumulated collagen gradually deposit into the network of collagen fiber. Most of collagen fibers are typically oriented almost parallel to the longitudinal direction of the original myofiber bundles [27], [28]. Other studies have shown that a small amount of collagen fiber is usually aligned at some oblique angle in the longitudinal direction [29]. Consequently, scarred myocardium consists largely of three-dimensional structural framework of fibrosis collagens to which discrete matrix components are attached [30], [31]. Our histological staining section clearly indicated that scarred myocardium was deficient in cellularity and mainly comprised discrete matrix deposition. Although increased extracellular volume percentage is mainly responsible for enlarged distribution of CAs, it cannot be neglected that capillary permeability is also the critical factor to affect the distribution of CA. Different from Gd-DTPA, distribution volume of P792 in scarred myocardium didn't differ from that in normal myocardium, indicating that the macromolecular CA was still retained within intravascular space. Therefore, the permeability of vessels within scarred myocardium is comparable to that of normal myocardium, permitting free diffusion of Gd-DTPA rather than P792 into enlarged interstitial space of scarred myocardium.

Delayed enhancement occurs for both acute (necrosis) and chronic (scar) myocardial infarction after the administration of Gd-DTPA [32]. The mechanism responsible for the delayed hyper-enhancement of myocardial infarction has been widely and extensively studied in prior investigations. However, most prior studies have focused more on acute infarction than chronic infarction. Delayed enhancement in acute infarction can be attributed to the increased extracellular space consequent to the loss of cell membrane integrity, which allows the contrast molecule to enter the myocyte intracellular space [33]. As infarction evolves, the necrotic myocytes were replaced by a dense fibrous scar with remodelled vessels. The pathological alterations of acute and chronic infarction differ considerably, thus the enhancement patterns in acute and chronic infarction might also be different. The delayed hyper-enhancement of chronic infarction is mostly due to remodelled microvessels supporting contrast transportation and the relatively large extracellular matrix of the developed scar allowing contrast accumulation.

In summary, MR contrast agents are able to enter the scarred myocardium as a consequence of newly formed collateral flow and remaining vessels of dilation within scar tissue. Microvascular permeability within scarred myocardium allows extracellular rather than intravascular CAs to leak into interstitial compartment. The striking decrease in cellularity and extensively discrete matrix deposition provide the enlarged extravascular space for extracellular CAs. Therefore, scarred myocardium exhibits hyperenhancement in Gd-DTPA enhanced T_1_ imaging, whereas administration of P792 causes the homogeneous enhancement in regions of scarred and normal myocardium.
